# Pressure-dependent supercritical CO₂ extraction of *Heliotropium arbainense*: phytochemical enrichment and enhanced antimicrobial, anti-inflammatory, and pro-apoptotic activities

**DOI:** 10.1186/s40643-026-01080-x

**Published:** 2026-05-18

**Authors:** Samy Selim, Samiah Hamad Al-Mijalli, Souzan Mohammed Kafy, Mohammed H. Alruhaili, Hattan S. Gattan, Mutasem S. Almehayawi, Mohammed Aladhadh, Emad M. Abdallah, Mohamed A. Amin

**Affiliations:** 1 Department of Clinical Laboratory Sciences, College of Applied Medical Sciences, Jouf University, Sakaka, Kingdom of Saudi Arabia; 2 Department of Biology, College of Sciences, Princess Nourah bint Abdulrahman University, P.O. Box 84428, 11671 Riyadh, Kingdom of Saudi Arabia; 3 Obstetrics and Gynecology Department, Faculty of Medicine, King AbdulAziz University, Jeddah, Kingdom of Saudi Arabia; 4 Department of Clinical Microbiology and Immunology, Faculty of Medicine, King Abdulaziz University, 21589 Jeddah, Kingdom of Saudi Arabia; 5 Department of Medical Laboratory Sciences, Faculty of Applied Medical Sciences, King Abdulaziz University, Jeddah, Kingdom of Saudi Arabia; 6 Special Infectious Agents Unit, King Fahad Medical Research center, King Abdulaziz University, Jeddah, Kingdom of Saudi Arabia; 7 Department of Emergency Medicine, Faculty of Medicine, King Abdulaziz University, Jeddah, Kingdom of Saudi Arabia; 8 Department of Food Science and Human Nutrition, College of Agriculture and Food, Qassim University, 51452 Buraydah, Kingdom of Saudi Arabia; 9 Department of Biology, College of Science, Qassim University, 51452 Buraydah, Kingdom of Saudi Arabia; 10 Botany and Microbiology Department, Faculty of Science (Boys), Al- Azhar University, Cairo, 11884 Egypt

**Keywords:** *Heliotropium arbainense*, Supercritical CO₂ extraction, Antimicrobial activity, Antioxidants, Biological efficacy, Apoptosis

## Abstract

Supercritical CO₂ extraction was applied to *Heliotropium arbainense* to evaluate how extraction pressure influences phytochemical composition and biological activity. Extractions of phenolic and flavonoid were performed at two pressures (200 and 550 bar), by using Supercritical CO₂. Phenolic and flavonoid compounds, were determined by HPLC analysis. Higher pressure extraction (550 bar) resulted in a greater extraction yield and a marked enrichment of phenolic and flavonoid compounds. Gallic acid (1496.01 µg/g) was the most prevalent component under SFE 1, followed by catechin (859.51 µg/g), rutin (824.55 µg/g), and coumaric acid (559.93 µg/g). Both extracts exhibited broad antimicrobial activity against Gram-positive and Gram-negative bacteria as well as *Candida albicans*, with consistently lower MIC and MBC/MFC values observed for the high-pressure extract. Cytotoxic evaluation against SKOV3 ovarian cancer cells revealed a clear dose-dependent reduction in cell viability. The calculated IC₅₀ values further confirm the greater cytotoxic potency of extract at 550 bar, with a lower IC₅₀ (153.04 ± 0.4 µg/mL) compared to extract at 200 bar (183.18 ± 2.29 µg/mL) against SKOV3 cells. However, flow cytometry revealed that extract at 200 bar produced slightly higher early apoptosis (43.79% vs. 43.39%) and greater necrosis (21.83% vs. 13.29%) in SKOV3 ovarian cells overall. Anti-inflammatory activity, assessed by protein denaturation inhibition, was observed for both extracts, with the lower-pressure extract showing slightly stronger inhibitory efficiency. Pressure-dependent supercritical CO₂ extraction significantly influenced the phytochemical richness and biological performance of *H. arbainense*. These findings highlight the importance of extraction conditions in maximizing the functional potential of plant-derived bioactive compounds and support further investigation of *H. arbainense* as a source of biologically active phenolics.

**Graphical abstract:**

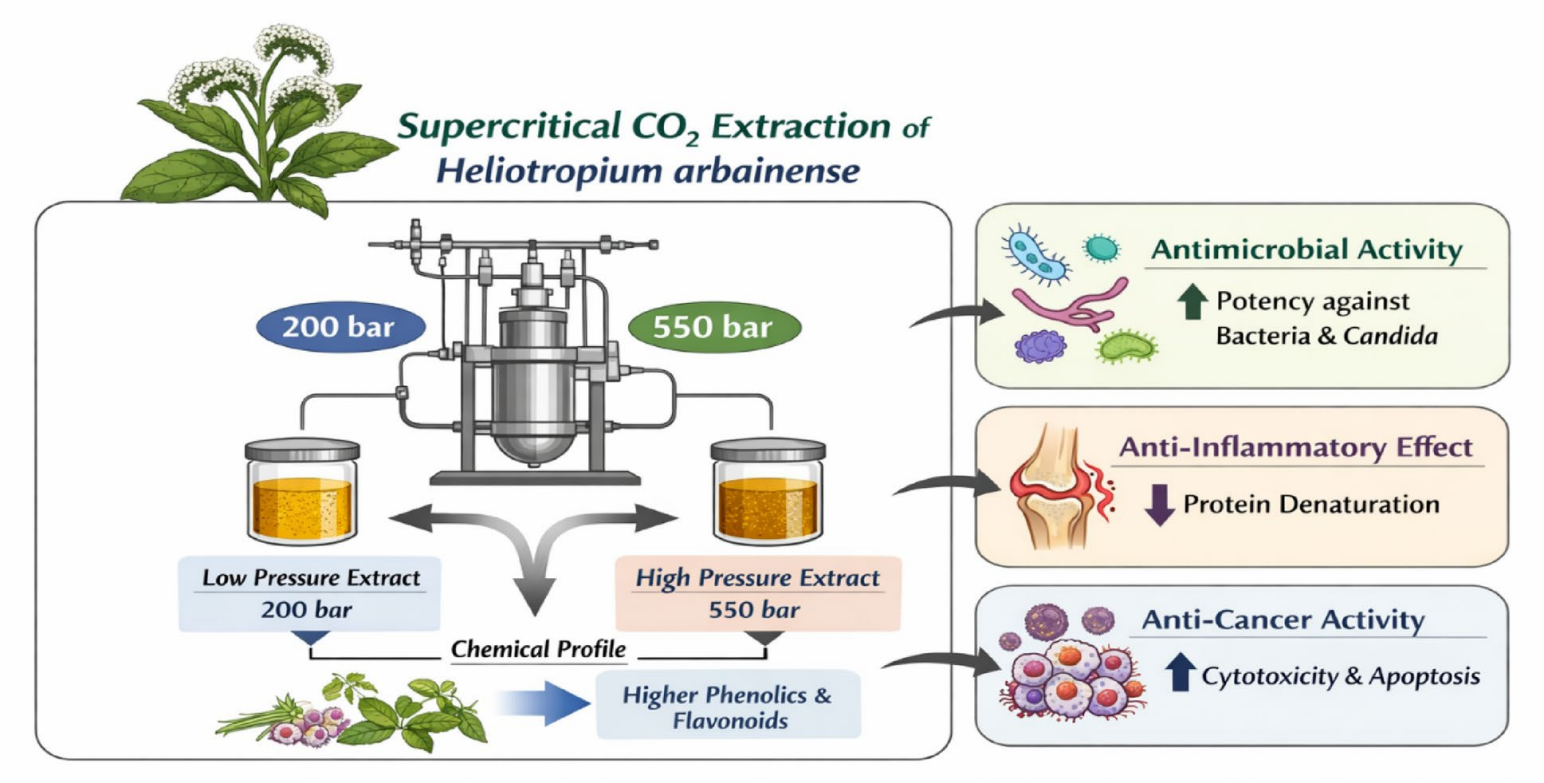

## Introduction

Plant extracts have received increasing attention for their wide industrial, nutritional, and medicinal applications, as medicinal plants remain a critical source of chemically diverse bioactive molecules and recent advances in green extraction technologies now enable their selective recovery with improved efficiency and biological relevance (Alawlaqi et al. 2023; Yıldırım et al. 2024). These natural products have strong antioxidant, antibacterial, anti-inflammatory, and anticancer activities because they are rich in bioactive phytochemicals such as phenolics, flavonoids, alkaloids, and essential oils (Selim et al. 2024; Alsolami et al. 2025). They are excellent prospects for creating safer and more potent substitutes for synthetic medications due to their wide range of biological functions. Because of their natural origin and low toxicity, plant extracts are widely used not only in medicine but also in food preservation, cosmetics, nutraceuticals, and functional drinks. The significance of plant-based bioactives has been further highlighted by growing interest in environmentally friendly and sustainable solutions, positioning them as promising resources for industrial and pharmaceutical innovation (Al-Rajhi et al. 2022; Bakri et al. 2024). However, for many medicinal plants, the influence of supercritical CO₂ extraction pressure on phytochemical composition and downstream biological activities remains insufficiently explored (Uwineza & Rukeshimana, 2020; Lioi et al. 2024). Addressing this gap, the present study evaluates pressure-dependent supercritical CO₂ extraction of *Heliotropium arbainense* and examines how extraction conditions shape its phytochemical profile and associated antimicrobial, anti-inflammatory, and pro-apoptotic activities.

According to Ghori et al. (2016), Heliotropium species have long been used in traditional medicine to treat conditions like rheumatism, gout, inflammation, skin ailments, irregular menstruation, and dangerous stings. The potential of Heliotropium species in phytochemistry, botany, pharmacology, and nutrition are immense. Terpenoids, flavonoids, and pyrrolizidine alkaloids are among the Heliotropium species’ active biochemical constituents. Fayed (2021) found that active components from different Heliotropium species have demonstrated significant biological activity, including antibacterial, antiviral, anticancer, anti-inflammatory, cytotoxic, phytotoxic, and wound-healing properties. Additionally, *Heliotropium arbainense* ethyl acetate and ether extracts shown definite antibacterial activity against *Clavibacter michiganensis*, according to Eid et al. (2023). Another species of Heliotropium genus, *H. subulatum* have demonstrated significant anti-cancer activity, antineoplastic inhibition and antioxidant properties (Singh et al., 2017). Comparable antimicrobial trends have been documented in other *Heliotropium* species. *H. indicum* is recognized for a wide spectrum of pharmacological attributes, including antimicrobial, antinociceptive, anti-ulcer, anti-glaucoma, anti-tuberculosis, antiplasmodial, and wound-healing activities (Pahuja et al. 2022).

Supercritical Fluid Extraction (SFE) has emerged as a significant green extraction technology that is extensively utilized for the recovery of bioactive compounds from natural sources (Almehayawi et al. 2024). Using supercritical CO₂ as the main solvent, SFE provides considerable advantages, including high extraction efficiency, selectivity, and the capability to maintain thermolabile compounds due to its low operating temperatures (Bazaid et al. 2025b). The adjustable solvating power of supercritical fluids facilitates precise control over the extraction of specific phytochemicals by modifying pressure and temperature, which allows for the targeted isolation of valuable components such as phenolics, flavonoids, essential oils, and lipophilic compounds. Moreover, SFE is environmentally sustainable, does not leave toxic solvent residues, and is in line with clean-label and sustainable processing trends. Due to these advantages, SFE is increasingly adopted in the pharmaceutical, food, cosmetic, and nutraceutical sectors for the production of high-purity extracts that offer improved safety and quality (Bazaid et al. 2025a; Qanash et al. 2025). Therefore, the current study aimed to evaluate the effect of supercritical CO₂ extraction pressure on the extraction yield, phytochemical composition, and biological activities of *Heliotropium arbainense*. Specifically, the study aimed to compare extracts obtained at different pressure levels in terms of their phenolic and flavonoid profiles, antimicrobial potency, anti-inflammatory activity, and cytotoxic and pro-apoptotic effects against SKOV3 ovarian cancer cells.

## Materials and methods

### Supercritical fluid extraction (SFE) of *H. arbainense*

The supercritical fluid extraction (SFE) of *H. arbainense* was performed using an ISCO-Sitec modified SFX 220 supercritical fluid extraction system. Dried leaves of *H. arbainense* (5.0 g per run) were subjected to CO₂-based supercritical extraction at two different pressure conditions (200 bar and 550 bar) while maintaining a constant temperature of 50 °C and an extraction duration of 40 min. After completion of extraction, the obtained extracts were collected, weighed, and stored at 4 °C in amber vials until further analysis. The variation in extraction yield at different pressures highlights the influence of pressure on CO₂ solubility and the efficiency of phytochemical recovery from *H. arbainense* leaves.

### HPLC method for determination of phenolic and flavonoid compounds in *H. arbainense*

The phenolic and flavonoid constituents in the extracts obtained at different supercritical fluid extraction pressures were analyzed using high-performance liquid chromatography (HPLC). Each extract was accurately weighed and dissolved in HPLC-grade methanol, mixed thoroughly, and filtered through a 0.22 μm membrane filter to remove any particulate matter. The clear filtrate was transferred into amber vials and injected directly into the HPLC system. Chromatographic separation was carried out on a reversed-phase C18 column (250 × 4.6 mm, 5 μm). The mobile phase comprised solvent A (0.1% formic acid in distilled water) and solvent B (acetonitrile), delivered using a gradient elution program tailored to achieve efficient separation of the target phenolic standards. The flow rate was maintained at 1.0 mL/min, and the injection volume was set at 20 µL for all samples and standards. The column temperature was kept constant at 30 °C to ensure reproducible retention. Detection was performed using a UV–Vis detector, monitoring wavelengths of 280 nm for phenolic acids and 320 nm for flavonoid compounds. Identification of individual peaks was achieved by comparing their retention times with those of pure reference standards analyzed under the same chromatographic conditions. Quantification was carried out using external calibration curves constructed for each standard, and the content of each compound was expressed as micrograms per gram of extract (Qanash et al. 2023).

### Antimicrobial activity of *H. arbainense* using the well microdilution method (MIC and MBC/MFC)

The antimicrobial activity of the extracts obtained at different supercritical fluid extraction pressures was assessed using the broth microdilution method. The tested microorganisms included *Bacillus subtilis* (ATCC 6633), *Staphylococcus aureus* (ATCC 6538), *Pseudomonas aeruginosa* (ATCC 90274), *Salmonella typhi* (ATCC 6539), *Candida albicans* (ATCC 10221), and *Fusarium oxysporum* (ATCC 46995). All strains were freshly cultured and adjusted to standardized inoculum densities of approximately 1 × 10⁶ CFU/mL for bacterial strains and 1 × 10⁵ CFU/mL for fungal strains.

Serial two-fold dilutions of each extract were prepared in sterile 96-well microplates using Mueller–Hinton broth for bacterial assays and Sabouraud broth for fungal assays, yielding final extract concentrations ranging from 1000 to 7.81 µg/mL. Each well was loaded with 100 µL of the extract dilution and 100 µL of the corresponding microbial suspension, resulting in a total volume of 200 µL per well. Growth control wells contained inoculated medium without extract, while sterility control wells contained uninoculated medium only. All experiments were conducted in triplicate.

Microplates were incubated at 37 °C for 24 h for bacterial strains and at 28 °C for 48 to 72 h for fungal strains (Al-Rajhi et al. 2025a). Following incubation, microbial growth was assessed by visual inspection for turbidity. The minimum inhibitory concentration (MIC) was defined as the lowest extract concentration that completely inhibited visible microbial growth compared with the growth control.

To determine the minimum bactericidal concentration (MBC) or minimum fungicidal concentration (MFC), 10 µL aliquots from wells showing no visible growth were subcultured onto the appropriate solid media (Al-Rajhi et al. 2024). Plates were incubated under the same conditions as the MIC assay, and the lowest extract concentration that produced no detectable colony growth was recorded as the MBC or MFC, confirming bactericidal or fungicidal activity rather than growth inhibition.

### Cytotoxicity assay of the *H. arbainense* extract on SKOV3 cells with apoptosis assay (annexin V–FITC/PI method)

SKOV3 human ovarian carcinoma cells were maintained in RPMI-1640 medium amended with 10% fetal bovine serum (FBS), 100 U/mL penicillin and 100 µg/mL streptomycin, and incubated at 37 °C in a humidified atmosphere containing 5% CO₂. Prior to treatment, cells were trypsinized and counted employing a hemocytometer. For viability testing, SKOV3 cells were seeded into 96-well plates at a density of 5 × 10³ cells per well in 100 µL complete medium and allowed to attach overnight. The test extract was prepared as a concentrated stock in dimethyl sulfoxide (DMSO) and diluted in culture medium immediately before use; final DMSO dose in all wells (including controls) did not exceed 0.5% (v/v). Cells were exposed to a series of extract concentrations (0, 31.25, 62.5, 125, 250, 500 and 1000 µg/mL) in triplicate wells. Parallel wells received vehicle control (0.5% DMSO) and a positive control anticancer agent (doxorubicin, included at a reference concentration series). Treatments were maintained for two days under standard culture conditions. Cell viability was quantified using the MTT assay. After the two days exposure, 10 µL of MTT reagent (5 mg/mL in PBS) was added to each well and plates were incubated for 3–4 h at 37 °C to allow formation of formazan crystals. The medium was then gently removed and 100 µL of DMSO was added to each well to dissolve the crystals (Qanash et al. 2023) Absorbance was read at 570 nm using a microplate reader with a reference wavelength of 630 nm. Background absorbance was subtracted from all readings.

Percentage toxicity (cell death) was estimated for each treated well employing the formula:

$${\mathrm{Toxicity}}(\% ){\text{ = }}\left( {{\text{1 - }}\frac{{{\mathrm{Absorbancesofextract}}}}{{{\mathrm{Absorbancesofcontrol}}}}} \right) \times {\mathrm{100}}$$

Dose–response curves were constructed from the percent toxicity values and IC₅₀ values (concentration producing 50% toxicity) were estimated.

SKOV3 cells were seeded in 6-well plates at 3 × 10^5^ cells/well and allowed to adhere overnight, then treated in biological triplicate with vehicle (0.5% DMSO), IC₅₀ concentration of the extract for 24 h. Following treatment, both floating and adherent cells were collected, combined and washed twice with cold PBS, pelleted at 300 × g for 5 min and fixed by adding ice-cold 70% ethanol dropwise to a cell suspension in PBS to a final volume of 1 mL cell suspension + 4 mL ethanol; samples were stored at − 20 °C for 2 h. Fixed cells were centrifuged, washed once with PBS to remove ethanol, then resuspended in 500 µL staining solution containing propidium iodide (50 µg/mL) and RNase A (100 µg/mL) (optionally 0.1% Triton X-100) and incubated in the dark at room temperature for 30 min to ensure RNA digestion and DNA staining. Prior to acquisition samples were passed through a 35 μm mesh to remove clumps; at least 10,000 single-cell events per sample were recorded on a flow cytometer using the PI channel with instrument settings and voltages established from unstained and single-stain controls; doublet discrimination was applied (PI-area vs. PI-width) and debris excluded by FSC/SSC gating. DNA histograms were modeled using a cell-cycle fitting algorithm to quantify G0/G1, S and G2/M fractions, and results are reported as mean ± SD from three independent experiments (Al-Rajhi et al. 2025b).

### Protein denaturation inhibition method for estimation of anti-inflammatory activity of *H. arbainense* extract

The anti-inflammatory potential of the extract was evaluated utilizing the protein denaturation inhibition assay. This technique evaluates the ability of the extract to protect proteins from heat-induced denaturation, a process linked to inflammatory responses. Different concentrations of the extract (1.56–200 µg/g) were prepared freshly prior to the experiment. Diclofenac sodium was used as the reference anti-inflammatory drug at the same dose range. A reaction mixture consisting of 0.2 mL of fresh egg albumin and 2.8 mL of phosphate buffer (pH 6.4) was prepared. To each tube, 2 mL of the test extract at the selected concentration was added, resulting in a total reaction volume of 5 mL. A control tube containing albumin and buffer without extract was run in parallel. All mixtures were incubated at 37 °C for 15 min, followed by heating at 70 °C for 5 min to induce protein denaturation. After cooling to room temperature, absorbance was recorded at 660 nm using a UV–Vis spectrophotometer. The percentage inhibition of protein denaturation was calculated using the following formula:

$${\mathrm{Inhibition}}(\% ){\text{ = }}\left( {\frac{{{\mathrm{A}}_{{{\mathrm{control}}}} {\text{ - A}}_{{{\mathrm{sample}}}} }}{{{\mathrm{A}}_{{{\mathrm{control}}}} }}} \right) \times {\mathrm{100}}$$

where $\:{A}_{\mathrm{control}}$= absorbance of the denatured protein without extract, $\:{A}_{\mathrm{sample}}$= absorbance of the sample containing extract.

### Statistical analysis

Minitab 18 (Minitab LLC, State College, PA, USA) was used to conduct statistical analyses in triplicate (*n* = 3) with a *p* < 0.05 threshold for statistical significance. To ascertain the difference between group means, the study employed Tukey’s Honest Significant Difference (HSD) Test for all post hoc comparison tests.

## Result and discussion

### Supercritical fluid extraction (SFE) of *H. arbainense* and phytochemical analysis via HPLC

**Fig. 1:**
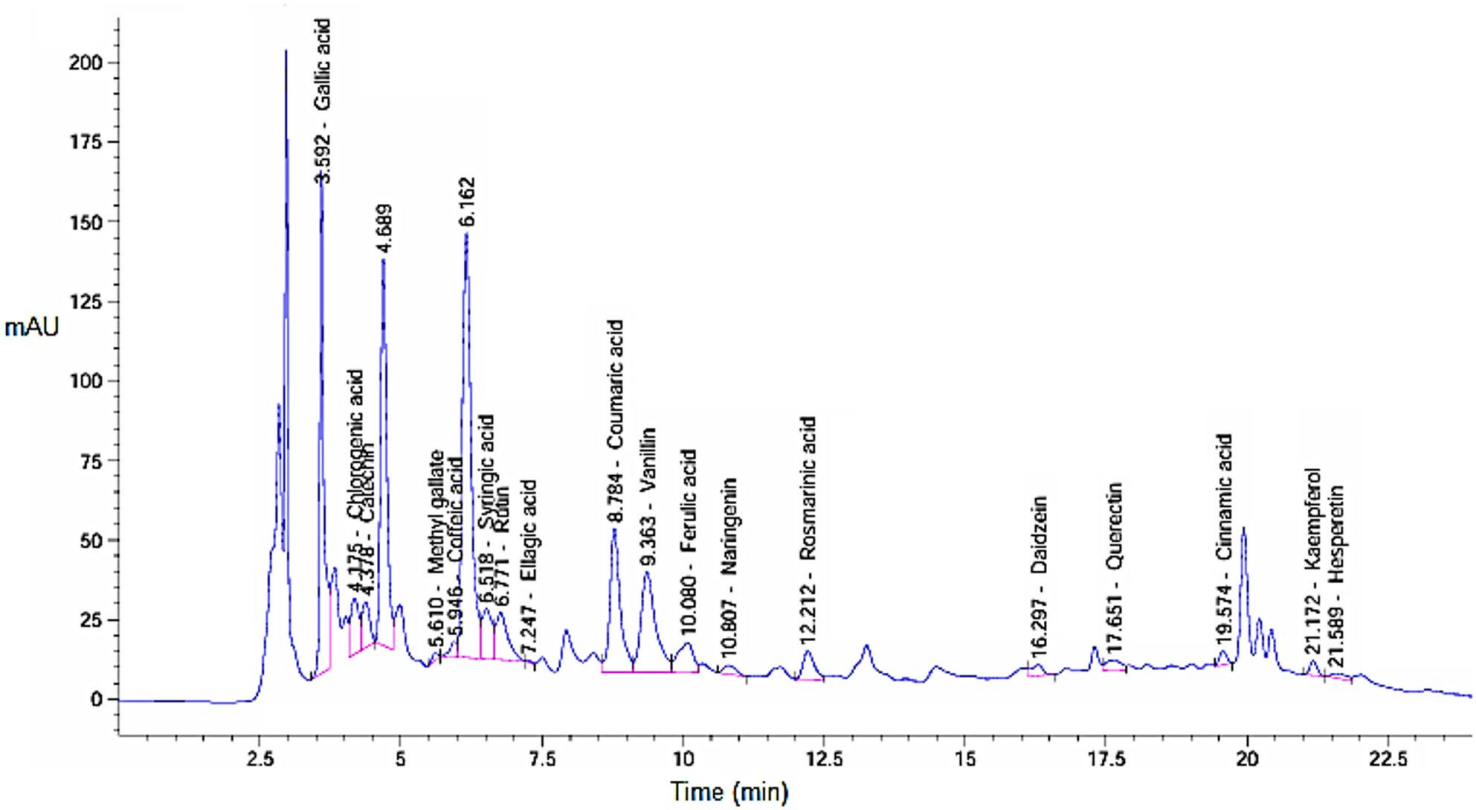
HPLC chromatogram illustrating retention times and peak intensities of phenolic and flavonoid compounds present in the extract of *H. arbainense* at pressure 550 bar (SFE1)

**Fig. 2:**
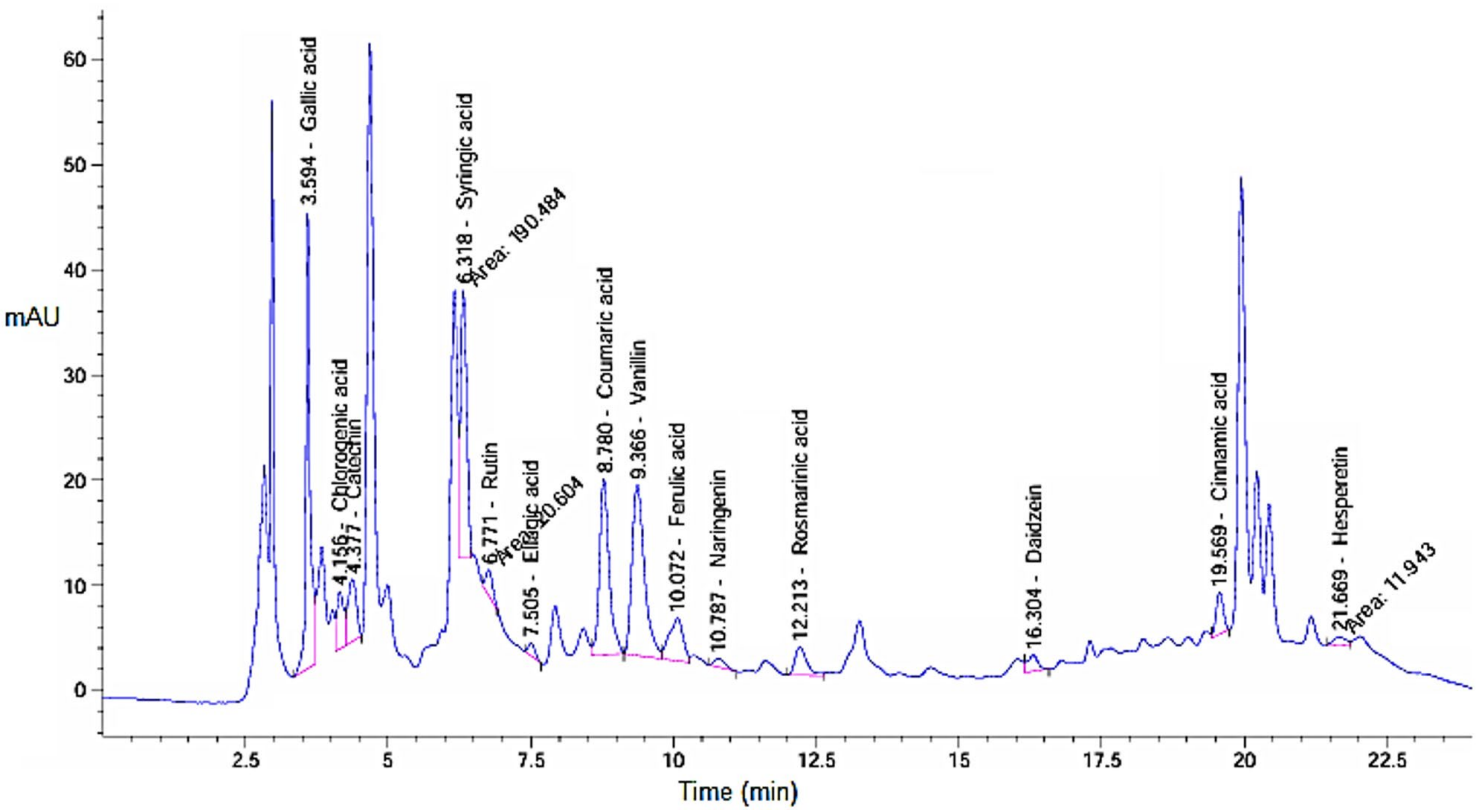
HPLC chromatogram illustrating retention times and peak intensities of phenolic and flavonoid compounds present in the extract of *H. arbainense* at pressure 200 bar (SFE2)

The results indicate that higher pressure (SFE1) produced a greater extract yield compared to lower pressure (SFE2), where the obtained yields were 0.234 g (4.68%) at pressure 550 bar (SFE1) and 0.163 g (3.26%) at pressure 200 bar (SFE2). This difference due to increased solubility of bioactive compounds in supercritical CO₂ at elevated pressures. Supercritical CO₂ was employed as the extraction solvent because it is non-toxic, environmentally friendly, and allows selective extraction of thermolabile and non-polar phytochemicals under relatively mild temperature conditions, minimizing thermal degradation. HPLC was used to analyze *H. arbainense* extracts obtained at two supercritical fluid extraction pressures including SFE 1 and SFE 2 as showed in Figs. 1 and 2, respectively as well as in Table 1. For each compound, the retention time (RT), peak area percentage, and concentration (µg/g) are reported, highlighting notable differences in yield between the two extraction conditions. Under SFE 1, gallic acid was the most abundant constituent, reaching 1496.01 µg/g, followed by catechin (859.51 µg/g), rutin (824.55 µg/g), and coumaric acid (559.93 µg/g). Vanillin and syringic acid also contributed meaningly with 517.30 µg/g and 329.49 µg/g, respectively. Several moderate-level compounds were detected, including ferulic acid (283.94 µg/g), rosmarinic acid (293.23 µg/g), and quercetin (201.77 µg/g). Less abundant compounds such as ellagic acid (10.17 µg/g) and cinnamic acid (22.47 µg/g) were also present. The total quantified phenolic content for SFE 1 reached 6449.65 µg/g. In contrast, the SFE 2 extract showed generally lower concentrations, with a total yield of 3965.15 µg/g. Gallic acid remained a major compound but decreased to 793.13 µg/g, while catechin and syringic acid recorded 719.09 µg/g and 609.73 µg/g, respectively. Vanillin and coumaric acid also showed notable amounts at 418.18 µg/g and 356.27 µg/g. Several constituents present in SFE 1- such as methyl gallate, caffeic acid, quercetin, and kaempferol-were absent in SFE 2, indicating reduced extraction efficiency at this pressure. Higher pressures greatly increase CO_2_’s density, which enhances its capacity to solubilize moderately polar chemicals, especially when co-extracted matrix components or weak intermolecular contacts aid their transfer. Since both caffeic acid and methyl gallate are moderately polar phenolic chemicals, their extraction under high pressure indicates that higher CO2 density improves their apparent solubility and transport (Janghel et al. 2015).

SFE has consistently been shown to improve the recovery of bioactive constituents, particularly phenolic and flavonoid compounds, across a wide range of medicinal plants. Alsalamah et al. (2025) reported that increasing extraction pressure during green cardamom processing led to a gradual increase in most phenolics detected via HPLC, with the highest concentration profile obtained at 300 bar. This pressure-dependent enrichment of phytochemicals is consistent with findings in other SFE-based studies. For instance, Bazaid et al. (2025a) demonstrated that *Thymus vulgaris* extracted at 30 MPa contained the greatest amounts of total phenolics, tannins, flavonoids, alkaloids, and saponins, and key phenolic acids such as kaempferol, rutin, syringic, rosmarinic, and caffeic acids. Similarly, SFE-CO₂ extraction of *Moringa peregrina* yielded markedly higher quantities of gallic acid, chlorogenic acid, vanillin and rutin at 25 MPa compared with lower or higher pressures, as shown by Qanash et al. (2025). Almehayawi et al. (2024) also highlighted that *Moringa oleifera* extracted at 25 MPa produced maximum yields of flavonoids, phenolics, tannins, alkaloids, and several phenolic acids, whereas extraction at 15 or 35 MPa resulted in significantly lower concentrations.

The present findings, together with the cited studies, underscore that SFE pressure significantly influences the extraction efficiency of phytoconstituents. The consistent observation across species indicates that intermediate-to-high pressures facilitate improved solubility and mass transfer efficiency of polar phenolic compounds in supercritical CO₂ systems, leading to superior phytochemical profiles.

**Table 1:** Phenols and flavonoid compounds of in the *H. arbainense* extract at different pressure levels

Detected constituents	SFE 1			SFE 2		
	RT	Area %	Conc. (µg/g)	RT	Area %	Conc. (µg/ g)
Gallic acid	3.592	13.709	1496.01	3.594	18.627	793.13
Chlorogenic acid	4.175	3.056	644.81	4.156	3.752	308.98
Catechin	4.378	2.458	859.51	4.377	5.271	719.09
Methyl gallate	5.610	0.287	24.54	5.633	0.00	0.00
Caffeic acid	5.946	0.662	59.63	5.992	0.00	0.00
Syringic acid	6.518	3.418	329.49	6.318	16.208	609.73
Rutin	6.771	3.766	824.55	6.771	1.753	149.78
Ellagic acid	7.247	0.059	10.17	7.505	0.842	56.24
Coumaric acid	8.784	10.182	559.93	8.780	16.603	356.27
Vanillin	9.363	10.148	517.30	10.16	21.025	418.18
Ferulic acid	10.080	3.260	283.94	11.83	5.972	202.97
Naringenin	10.807	0.698	101.63	17.55	0.947	53.79
Rosmarinic acid	12.212	2.191	293.23	19.13	3.369	175.90
Daidzein	16.297	0.841	72.43	16.304	1.636	54.95
Querectin	17.651	1.035	201.77	17.598	0.00	0.00
Cinnamic acid	19.574	0.689	22.47	19.569	2.981	37.91
Kaempferol	21.172	0.718	110.91	20.842	0.00	0.00
Hesperetin	21.589	0.524	37.32	21.669	1.016	28.24
Total content			6449.65			3965.15

### Biological activities of *H. arbainense* extracts obtained through SFE1 and SFE2

**Fig. 3:**
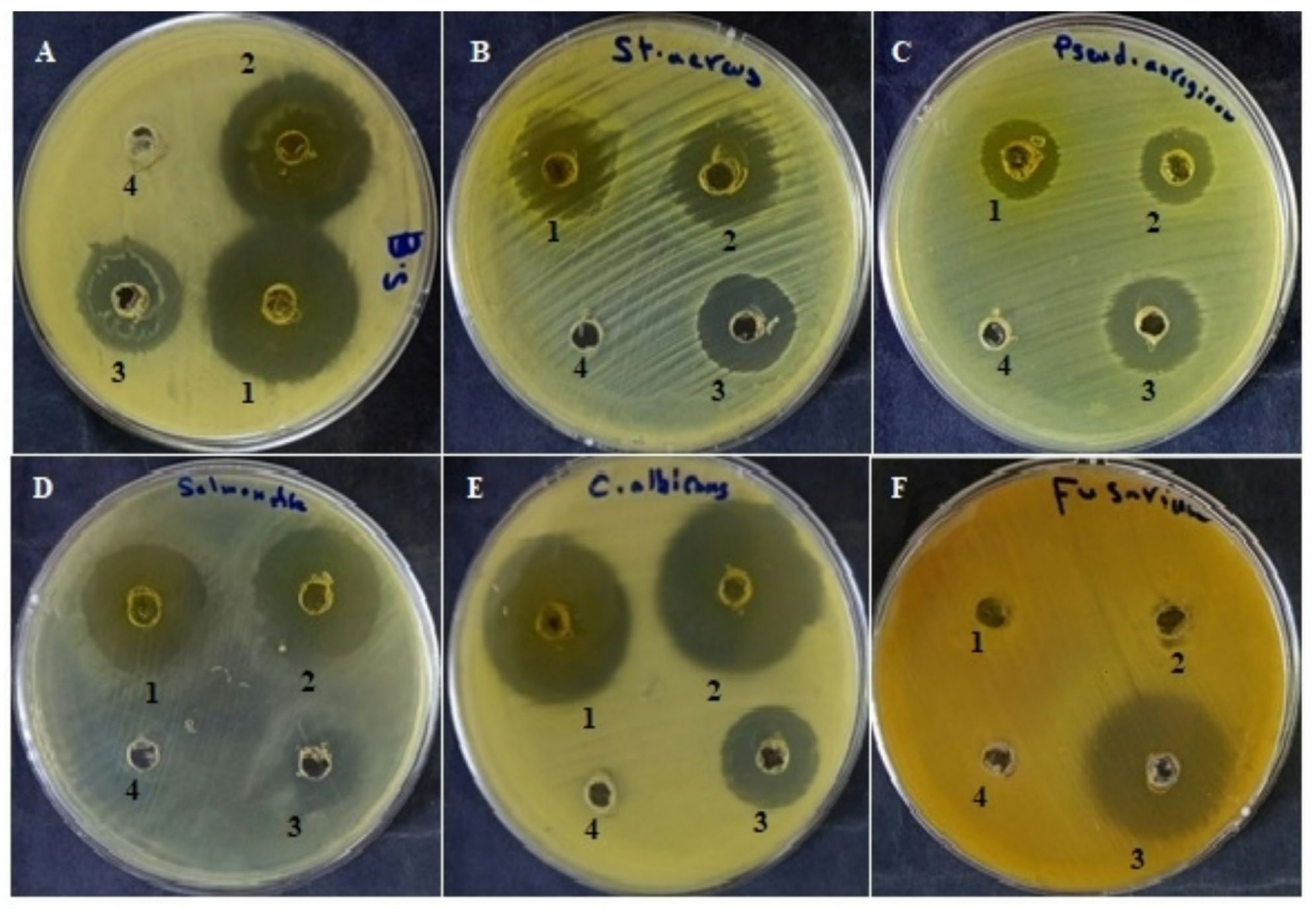
Antimicrobial activity of*H. arbainense*extracts (SFE1 and SFE2) against tested microorganisms. **A***B. subtilis*, **B***Staphylococcus aureus*, **C***Pseudomonas aeruginosa*, **D***Salmonella*spp.,**E***Candida albicans*, **F** *Fusarium oxysporum.*Wells: 1 = SFE1, 2 = SFE2, 3 = Antibiotic/antifungal control, 4 = Negative control

The antimicrobial activity of *H. arbainense* extracts obtained by SFE1 and SFE2 against the tested microbial strains is summarized in Table 2 and illustrated in Fig. 3. Overall, SFE1 consistently produced larger inhibition zones against most microorganisms compared with SFE2. The strongest antibacterial activity was observed against *Bacillus subtilis*, for which SFE1 produced an inhibition zone of 32 ± 0.84 mm, followed by SFE2 (29 ± 0.54 mm); both values were notably higher than that of the control (23 ± 0.64 mm). In agreement with these results, both extracts exhibited low MIC values (15.62 µg/mL), indicating high susceptibility of *B. subtilis* to the extracts.

Moderate antibacterial activity was recorded against *Staphylococcus aureus*. SFE1 and SFE2 produced inhibition zones of 19 ± 0.92 mm and 17 ± 0.32 mm, respectively, whereas the control showed a slightly larger zone (20 ± 0.45 mm). MIC values demonstrated a stronger inhibitory effect for SFE1 (31.25 µg/mL) compared with SFE2 (62.5 µg/mL), with MBC values following a similar trend. In contrast, both extracts exhibited lower activity against *Pseudomonas aeruginosa* than the control, with inhibition zones of 16 ± 0.51 mm for SFE1 and 14 ± 0.23 mm for SFE2. The corresponding MIC and MBC values (31.25–125 µg/mL) reflect the reduced susceptibility of this pathogen.

Both extracts showed pronounced antibacterial effects against *Salmonella typhi*. SFE1 and SFE2 generated inhibition zones of 26 ± 0.58 mm and 25 ± 0.29 mm, respectively, which were significantly larger than the control (15 ± 0.39 mm). The low MIC values (15.62 µg/mL), together with moderate MBC values, confirm the strong antibacterial activity of the extracts against this organism. For the fungal strain *Candida albicans*, SFE1 and SFE2 exhibited potent antifungal activity, producing inhibition zones of 31 ± 0.23 mm and 30 ± 0.54 mm, respectively, both exceeding that of the control (20 ± 0.92 mm). The corresponding MIC and MBC values (15.62–31.25 µg/mL) further support the strong fungistatic and fungicidal effects of the extracts. In contrast, neither extract showed activity against *Fusarium oxysporum*, as no inhibition zones or MIC/MFC values were detected, indicating complete resistance.

The antimicrobial efficacy of plant extracts obtained by supercritical fluid extraction is closely linked to extraction pressure and the resulting phytochemical enrichment. Alsalamah et al. (2025) reported that green cardamom extracted at 300 bar exhibited the highest antimicrobial potency, with pronounced inhibition against *Candida albicans* and *Bacillus subtilis*, as well as strong antibiofilm and anti-hemolytic activities. Similarly, Bazaid et al. (2025a) demonstrated that *Thymus vulgaris* extracted at 30 MPa produced the largest inhibition zones and the lowest MIC and MBC values against a broad range of bacterial and fungal pathogens. Accumulating evidence also highlights the broad antimicrobial potential of species within the *Heliotropium* genus. Mekky et al. (2025) showed that methanolic extracts of *H. arbainense* aerial floral parts possess notable antibacterial activity, particularly against *Klebsiella pneumoniae* and *Staphylococcus aureus*, which was associated with their rich phytochemical content, including flavonoids, tannins, and phenolic compounds. Tannins are known to exert membrane-stabilizing and anti-inflammatory effects (Subramanya et al. 2018), while flavonoids contribute antioxidant and anti-inflammatory activities (Al-Khayri et al. 2022). In addition, extracts of *H. strigosum* have demonstrated antifungal and antibacterial activity against several clinically relevant pathogens (Hussain et al. 2010)d *bacciferum* extracts have been reported to inhibit multiple phytopathogens (Abdelkhalek et al. 2024).

Despite these reports, the present study showed that SFE-derived extracts of *H. arbainense* were inactive against *Fusarium oxysporum*. This discrepancy may be attributed to differences in extraction method, phytochemical composition, plant part used, or pathogen strain. Collectively, our findings support previous evidence that carefully regulated supercritical fluid extraction conditions enhances antimicrobial activity by concentrating bioactive phenolic and flavonoid compounds. The enhanced antimicrobial efficacy observed at 550 bar underscores the critical interplay between extraction conditions, phytochemical composition, and biological activity.

**Table 2:** Antimicrobail action (mm) & MIC and MBC (µg/ml) levels of *H. arbainense* extract via SFE1 and SFE2

Tested microbes	Zone of inhibition (mm)			MIC/MBC (µg/ml)			
	SFE 1	SFE 2	Control	SFE 1	SFE 2	SFE 1	SFE 2
*B. subtilis*	32 ± 0.84a	29 ± 0.54b	23 ± 0.64c	15.62	15.62	15.62	31.25
*S. aureus*	19 ± 0.92a	17 ± 0.32b	20 ± 0.45a	31.25	62.5	31.25	125
*P. aeruginosa*	16 ± 0.51b	14 ± 0.23c	21 ± 1.05a	31.25	62.5	31.25	125
*S. typhi*	26 ± 0.58a	25 ± 0.29a	15 ± 0.39b	15.62	15.62	31.25	125
*C. albicans*	31 ± 0.23a	30 ± 0.54a	20 ± 0.92b	15.62	15.62	31.25	31.25
*F. oxysporum*	NA	NA	28 ± 0.23a	–	–	–	–

**Fig. 4:**
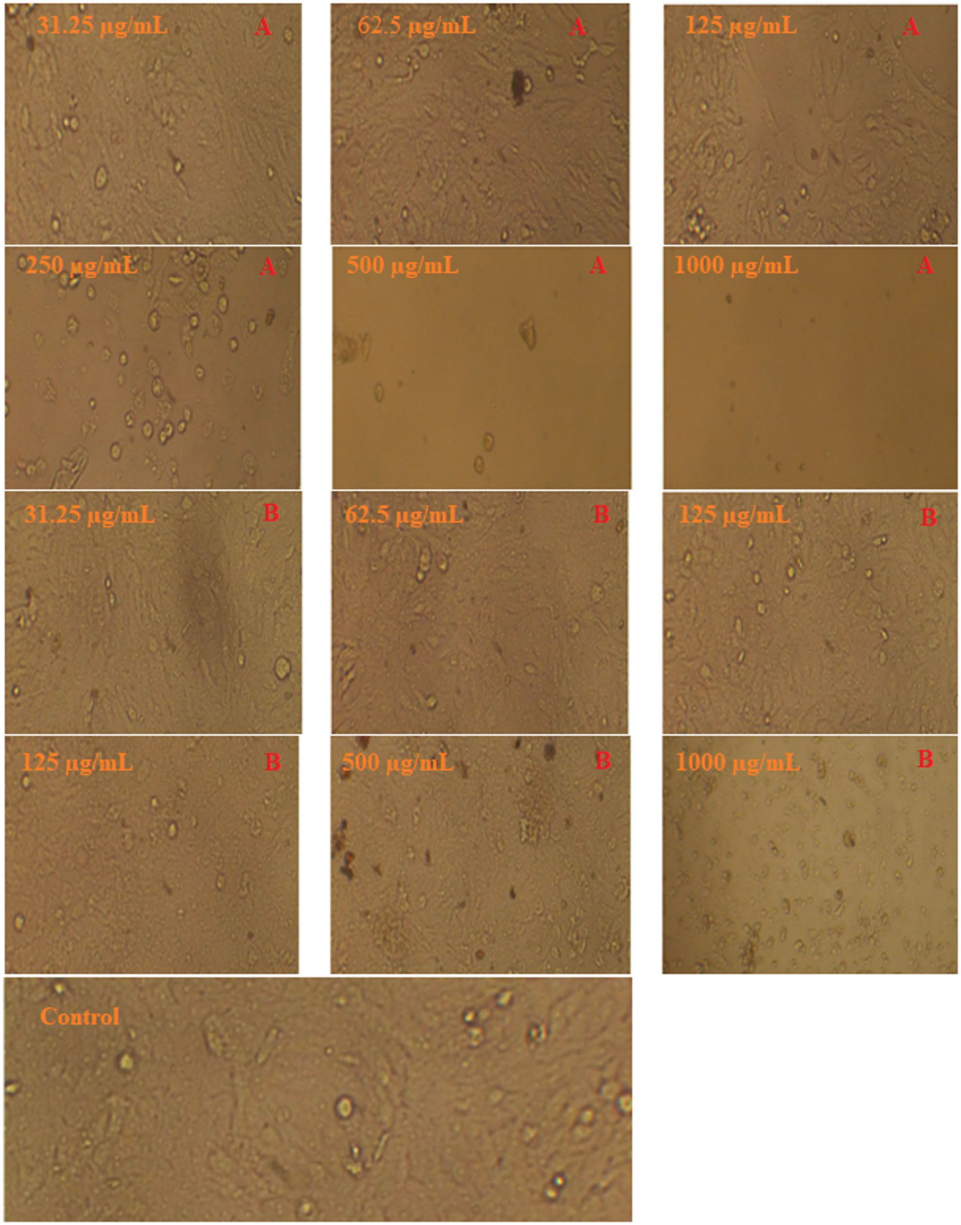
Morphological changes of SKOV3 exposed to different doses of *H. arbainense* extract using SFE 1 (**A**) and SFE 2 (**B**) against SKOV3 . Magnification 10 X

The cytotoxic effects of *H. arbainense* extract obtained using two supercritical fluid extraction conditions (SFE 1 and SFE 2) against SKOV3 ovarian cancer cells were reported (Table 3). SKOV3 is a well-known human epithelial ovarian carcinoma model that is useful for assessing possible anticancer options because of its aggressive phenotype, high proliferation capability, and partial resistance to traditional chemotherapeutic drugs (Ma et al. 2010). The toxicity (%) was evaluated at concentrations ranging from 0 to 1000 µg/mL. At the lowest concentration (31.25 µg/mL), both extracts exhibited minimal cytotoxicity; however, SFE 2 showed slightly higher toxicity (0.82%) compared to SFE 1 (0.21%). As the dose increased to 62.5 µg/mL, toxicity increased significantly, particularly in SFE 1, which reached 3.96%, exceeding the effect of SFE 2 (2.06%). A clear dose-dependent cytotoxic response was observed, with marked increases at higher concentrations. At 125 µg/mL, SFE 1 produced 43.47% toxicity, notably higher than SFE 2 (30.35%). This trend continued at 250 µg/mL (92.44% for SFE 1 vs. 74.59% for SFE 2) and 500 µg/mL (97.17% vs. 84.21%). At the highest concentration (1000 µg/mL), both extracts exhibited strong cytotoxicity, though SFE 1 remained slightly more potent (97.22%) than SFE 2 (95.99%). The calculated IC₅₀ values further confirm the greater cytotoxic potency of SFE 1, with a lower IC₅₀ (153.04 ± 0.4 µg/mL) compared to SFE 2 (183.18 ± 2.29 µg/mL). These results indicate that the extract obtained under SFE 1 conditions exhibits stronger anticancer activity against SKOV3 cells. The accompanying micrographs (Fig. 4) illustrate dose-dependent morphological alterations in SKOV3 cells following exposure to increasing concentrations of the extract. Cells treated with SFE 1 (A) and SFE 2 (B) show progressive structural deterioration, loss of adherence, cell shrinkage, and membrane disintegration at higher doses, consistent with the quantitative cytotoxicity data. Other species of *Heliotropium* caused death of cancer ells, for example Fayed et al. (2022) investigated the anticancer potential of the methanolic extract of *H. ramosissimum* across a panel of seven cell lines, including A-375, Colo-205, HepG-2, HeLa, and H-460. Their findings demonstrated a selective cytotoxic action, with the strongest effect observed against the Colo-205 colon cancer cells, while sparing normal cells. Mechanistic analysis indicated that the extract triggered both apoptotic and necrotic pathways in Colo-205 cells. Additionally, chlorogenic acid that existed in the extract of *H. arbainense* as detected by HPLC, was utilized in the suppress of human colon cancer cell growth via induction of cell-cycle arrest and promotion of apoptosis, as reported by Sadeghi Ekbatan et al. (2018).

**Fig. 5:**
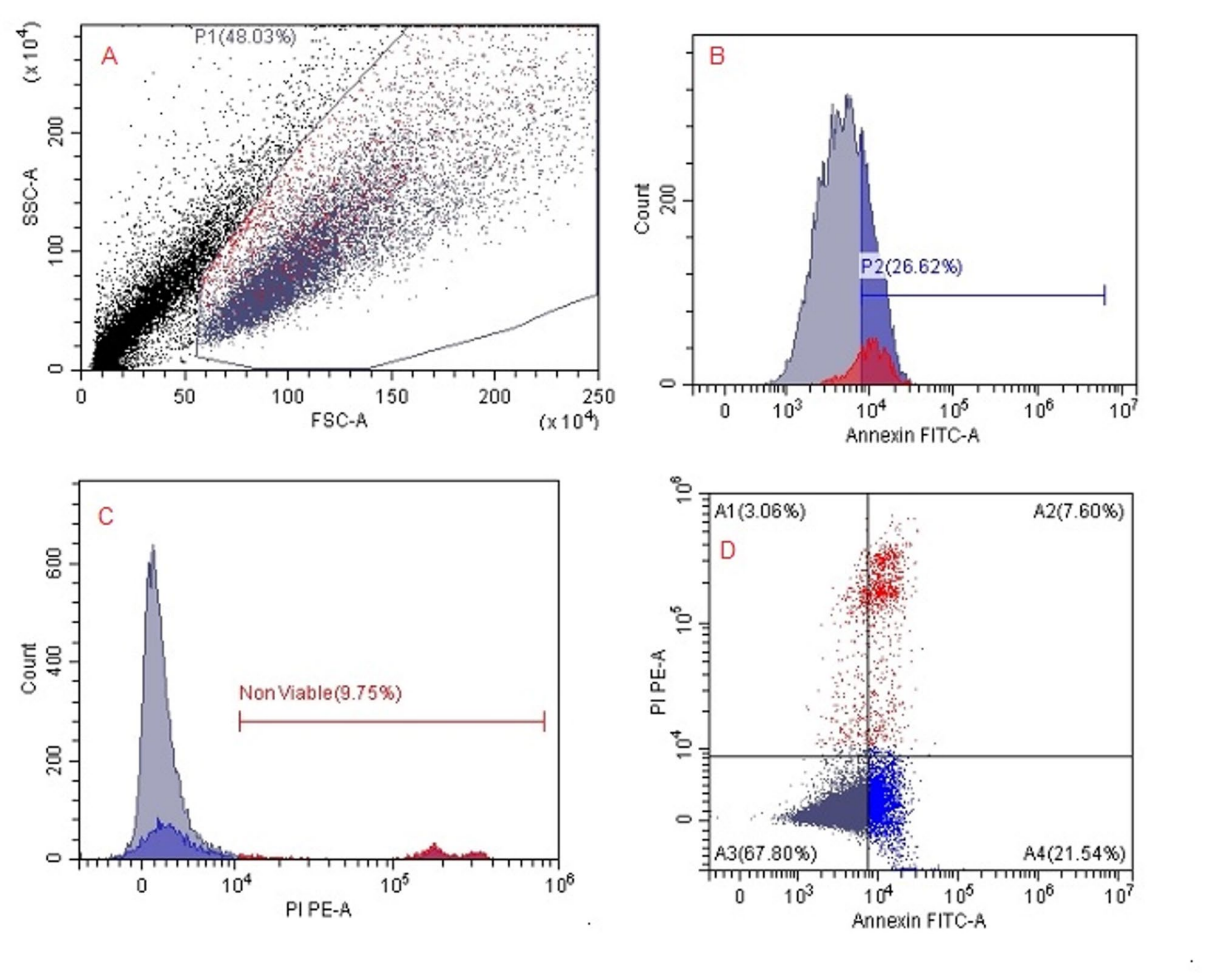
Flow cytometric analysis of apoptosis and viability in untreated SKOV3 cells. **A** Gating of the main cell population: Forward scatter (FSC-A) versus side scatter (SSC-A) plot showing the distribution and selection of viable untreated SKOV3 cells (P1). **B** Annexin V–FITC histogram: Untreated cells exhibit minimal Annexin V binding, indicating a very low percentage of early apoptotic cells. **C** PI histogram: Propidium iodide staining reveals a small proportion of PI-positive cells, representing naturally occurring non-viable cells in the culture. **D** Annexin V–FITC/PI dual-staining dot plot: Quadrant analysis demonstrates predominantly viable cells, with minimal early apoptosis (Annexin V–positive), late apoptosis/necrosis (dual-positive), or necrosis (PI-positive) populations, reflecting normal baseline cell viability

**Fig. 6:**
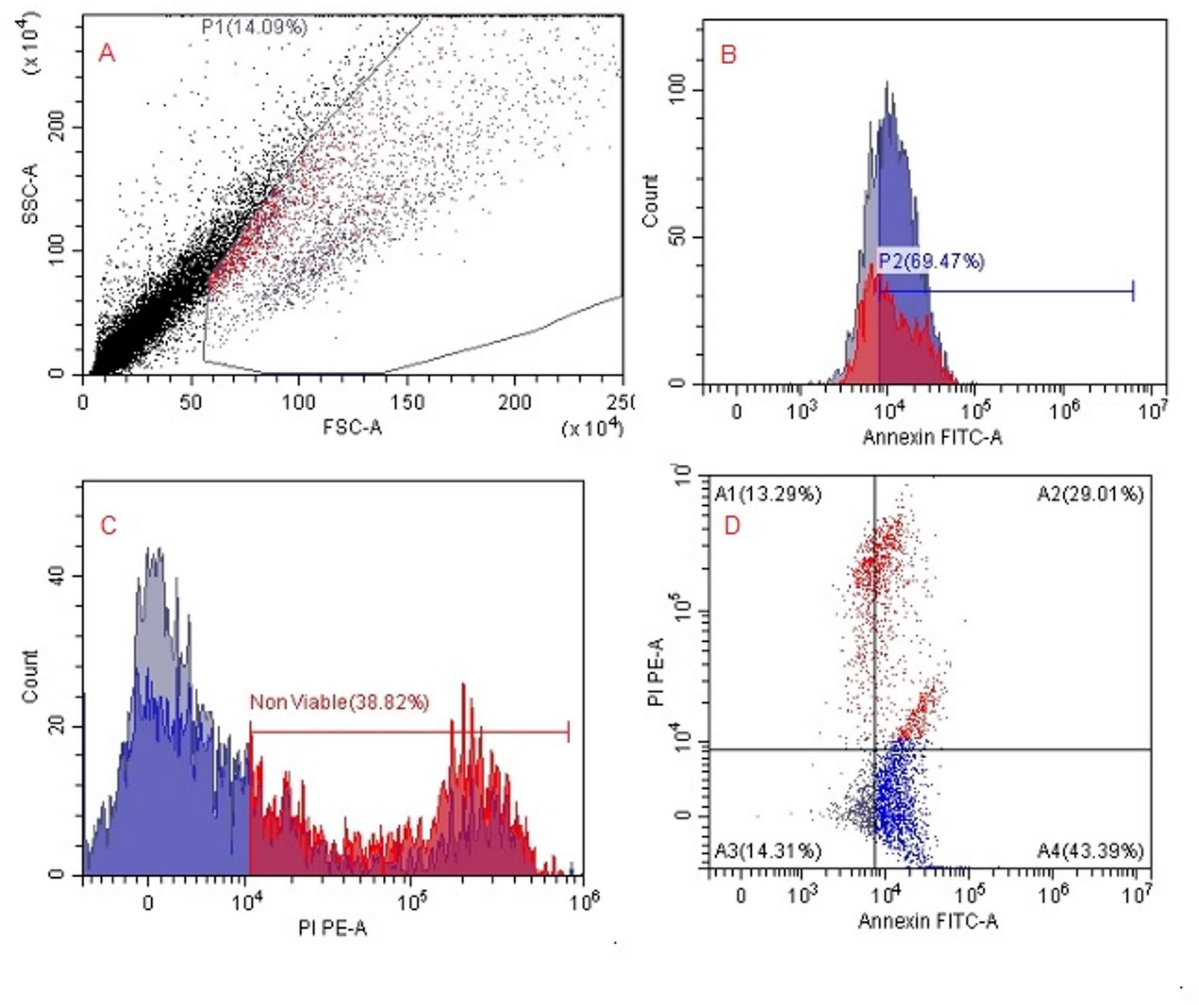
Flow cytometric analysis of apoptosis and cell death in SKOV3 cells treated with *H. arbainense* extract obtained using SFE 1. **A** Cell population gating: FSC-A versus SSC-A plot showing the gated SKOV3 cell population (P1 = 14.09%) after treatment, indicating reduced viable cell density compared to untreated cells. **B** Annexin V–FITC histogram: A marked increase in Annexin V–positive cells is observed, with 69.47% of the treated population falling within the apoptotic region (P2), reflecting strong **C** Propidium iodide (PI) histogram: PI staining reveals a high percentage of membrane-compromised, non-viable cells (38.82%), demonstrating substantial loss of viability following exposure to the extract. **D** Annexin V–FITC/PI dual-staining dot plot: Quadrant analysis identifies necrotic cells (A1, 13.29%), late apoptotic/necrotic cells (A2, 29.01%), viable cells (A3, 14.31%), and early apoptotic cells (A4, 43.39%). The distribution shows a dominant apoptotic profile, confirming that SFE 1 extract triggers both early and late apoptotic pathways in SKOV3 cells. induction of early apoptosis

**Fig. 7:**
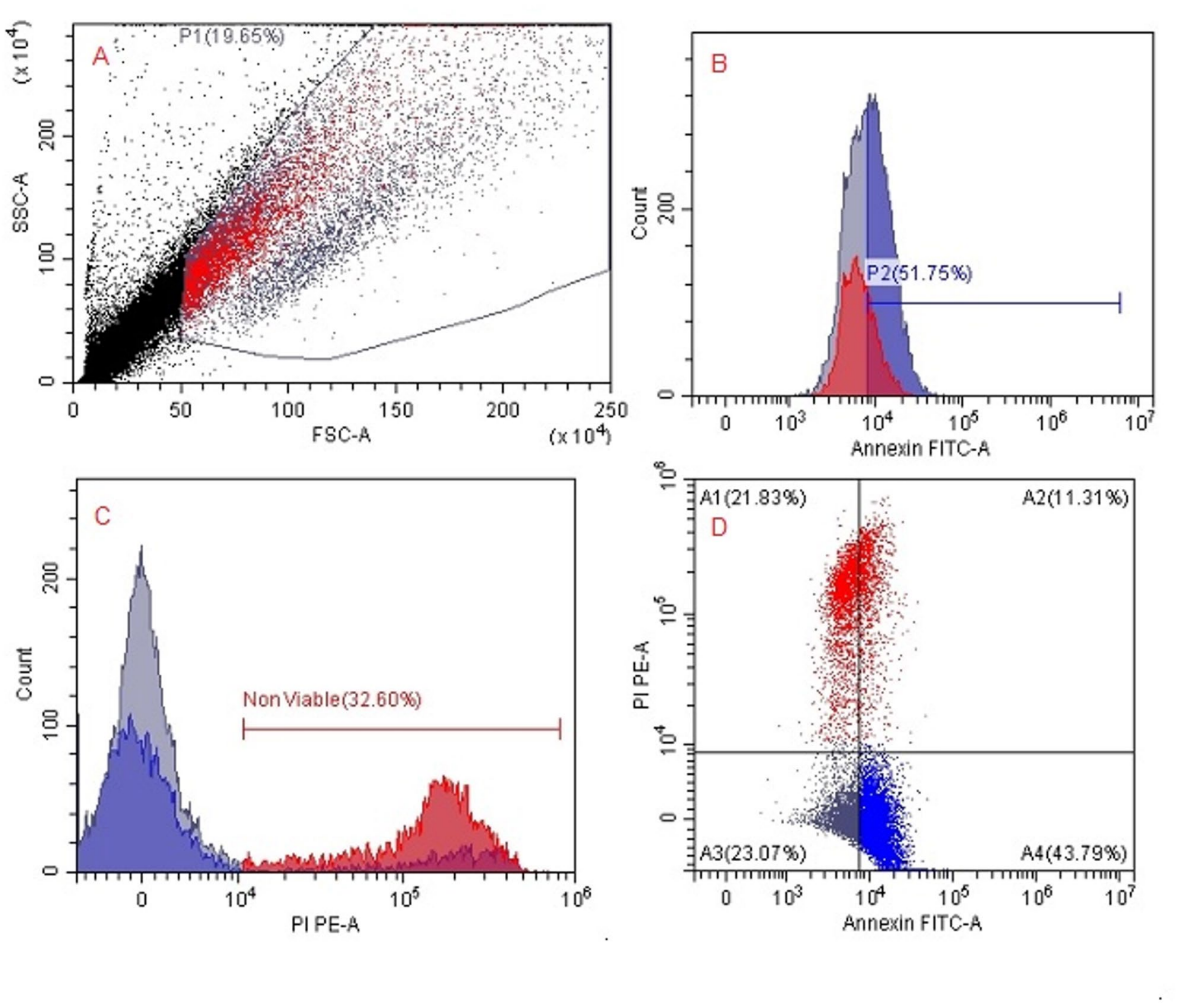
Flow cytometric analysis of apoptosis and cell death in SKOV3 cells treated with*H. arbainense*extract obtained using SFE-2. **A** Forward scatter (FSC-A) versus side scatter (SSC-A) dot plot showing the gated SKOV3 cell population (P1). **B** Annexin V–FITC histogram displaying the percentage of Annexin-positive cells (early apoptotic population, P2). **C** Propidium iodide (PI) histogram illustrating the proportion of non-viable/late apoptotic or necrotic cells. **D** Annexin V–FITC versus PI dual-parameter dot plot demonstrating the distribution of viable cells (A4), early apoptotic cells (A1), late apoptotic cells (A2), and necrotic cells (A3). Together, these profiles confirm that SFE-2 extract induces marked apoptosis and loss of viability in SKOV3 ovarian cancer cells

Flow cytometric quantification of apoptotic and viable SKOV3 cell populations after treatment with *H. arbainense* extract obtained using SFE-1 and SFE-2 were recorded (Table 4; Figs. 5, 6 and 7). The outcomes detect the distribution of SKOV3 cells across the four standard Annexin V/PI quadrants, representing viable (A3), early apoptotic (A4), late apoptotic (A2), and necrotic (A1) populations. In untreated control cells, the majority of events (67.80% parent) were localized in the viable quadrant (A3), with only minor fractions undergoing early apoptosis (A4, 21.54%), late apoptosis (A2, 7.60%), or necrosis (A1, 1.47%). Treatment with SFE-1 resulted in a substantial shift toward apoptosis, particularly early apoptotic cells (A4, 43.39%) and late apoptotic/necrotic cells (A2, 29.01%), accompanied by a pronounced reduction in viable cells (A3, 14.31%). SFE-2 treatment further intensified apoptotic responses, producing the highest percentage of early apoptotic cells (A4, 43.79%) and an increased late apoptotic population (A2, 11.31%), while viable cells decreased markedly (A3, 23.07%). Generally, both extracts induced significant apoptosis in SKOV3 cells, with SFE-2 showing a slightly stronger effect on early apoptosis and SFE-1 producing higher late apoptosis/necrosis.

**Table 3:** Toxicity of different doses of *H. arbainense* extract using SFE 1 and SFE 2 against SKOV3.

Concentration (µg/mL)	Toxicity (%)		HSD (5%)
	SFE 1	SFE 2	
0.0	0.00 ± 0.00	0.00 ± 0.00	0.00
31.25	0.21 ± 0.06b	0.82 ± 0.09a	0.18
62.5	3.96 ± 0.18a	2.06 ± 0.34b	0.68
125	43.47 ± 1.03a	30.35 ± 2.19b	2.98
250	92.44 ± 2.03a	74.59 ± 1.41b	4.36
500	97.17 ± 1.52a	84.21 ± 2.41b	3.65
1000	97.22 ± 0.35a	95.99 ± 0.25a	2.95
IC_50_ µg/mL	153.04 ± 0.4b	183.18 ± 2.29a	4.85

Data represent means ± standard error (*n* = 3). Significant differences (*P* ≤ 0.05) are indicated by different lowercase letters in the same column

**Table 4:** Flow cytometric quantification of apoptotic and viable SKOV3 cell populations after treatment with *H. arbainense* extract obtained using SFE-1 and SFE-2

Population	Events			Total %			Parent %		
	Control	SFE1	SFE2	Control	SFE1	SFE2	Control	SFE1	SFE2
A2	890	882	1111	3.65	4.09	2.22	7.60	29.01	11.31
A1	358	404	2145	1.47	1.87	4.29	1.47	13.29	21.83
A3	7939	435	2267	32.57	2.02	4.53	67.80	14.31	23.07
A4	2522	1319	4303	10.35	6.11	8.61	21.54	43.39	43.79

The anti-inflammatory activity of *H. arbainense* extract obtained using two supercritical fluid extraction conditions (SFE1 and SFE2), assessed through the inhibition of protein denaturation at various doses (Table 5). Both extract types (SFE1 and SFE2) showed a clear dose-dependent enhancement in anti-inflammatory activity. SFE1 consistently displayed higher inhibition percentages compared to SFE2 at all concentrations. At the lowest dose (1.56 µg/g), SFE1 achieved 25.0 ± 1.01%, while SFE2 recorded 34.0 ± 1.36%, and these values progressively increased with higher doses. The highest activity was noted at 200 µg/g, where SFE1 reached 88.8 ± 1.02%, and SFE2 showed 90.7 ± 1.97%, approaching the activity of the standard drug. Also, as the dose increased, diclofenac sodium demonstrated a dose-dependent improvement in protein denaturation inhibition, reaching a maximum of 95.8 ± 0.65% at 200 µg/g. The IC₅₀ values highlight the relative potency of the treatments. Diclofenac sodium showed the lowest IC₅₀ (2.55 ± 0.84 µg/g), indicating the highest efficacy. Among the extracts, SFE2 demonstrated superior potency with an IC₅₀ of 5.29 ± 0.32 µg/g, followed by SFE1 with 9.63 ± 1.04 µg/g. According to Fayed et al. (2021), species of *Heliotropium* exhibit notable anti-inflammatory activity. Their hepatoprotective potential has also been documented in both traditional medicine and recent scientific investigations, largely attributed to bioactive constituents with potent antioxidant and anti-inflammatory actions. These compounds help alleviate oxidative stress, support liver function, and modulate detrimental biochemical indicators (Alolga et al. 2022). Gallic acid, which was identified in our extract, is known for its strong anti-inflammatory potential, primarily through the suppression of pro-inflammatory cytokines and key inflammatory enzymes. This mechanism highlights its relevance as a promising candidate for managing inflammation-related disorders. Additionally, gallic acid has been reported to possess anticancer activity by inhibiting tumor cell proliferation and inducing programmed cell death, as noted by Hadidi et al. (2024). The superior anti-inflammatory potency of SFE2, despite its 39% lower total phenolic content, highlights that biological activity is governed by compositional specificity rather than bulk phenolic abundance. This is clearly reflected in its IC₅₀ value, compared to IC₅₀ of SFE1, indicating a more efficient inhibition of protein denaturation. Mechanistically, this enhanced activity can be attributed to the selective enrichment of key bioactive constituents in SFE2. Notably, syringic acid is markedly elevated in SFE2 (609.73 µg/g) compared to SFE1 (329.49 µg/g). Ellagic acid, detected exclusively in SFE2 (56.24 µg/g) but 10.17 µg/g in SFE1, may further contribute to the enhanced anti-inflammatory activity through complementary mechanisms. This compound has been widely reported to exert anti-inflammatory effects by attenuating COX-2-mediated inflammatory responses and reducing the exacerbation of inflammation in experimental models (Cornélio Favarin et al. 2013). The presence of ellagic acid uniquely in SFE2 therefore provides an additional mechanistic layer, supporting the superior inhibition of protein denaturation observed for this extract. Its action, combined with the elevated levels of syringic acid, suggests a synergistic modulation of key inflammatory pathways, including enzyme inhibition and stabilization of protein structure under stress conditions. Consistent with the observed compositional differences, although catechin is lower in SFE2, it remains sufficiently abundant to contribute to bioactivity, while the marked enrichment of syringic acid likely plays a dominant and synergistic role in enhancing anti-inflammatory potency despite the lower total phenolic content (Shikha et al. 2024; Zhao et al. 2025).

**Table 5:** Anti-inflammatory Potential of *H. arbainense* extract via SFE1 and SFE2 at Different doses

Dose (µg/g)	Inhibition of protein denaturation (%)			HSD (5%)
	SFE2	SFE1	Diclofenac sodium	
0.0	0.00 ± 0.00	0.00 ± 0.00	0.00 ± 0.00	0.00
1.56	34.0 ± 1.36b	25.0 ± 1.01c	41.1 ± 1.03a	4.36
3.125	43.8 ± 2.32b	35.4 ± 0.96c	50.8 ± 1.25a	3.89
6.25	51.9 ± 1.65b	45.3 ± 2.32c	61.2 ± 1.36a	6.49
12.5	61.2 ± 3.32b	53.3 ± 1.85c	71.0 ± 0.94a	5.85
25	70.5 ± 0.66b	62.9 ± 0.56c	81.2 ± 1.52a	3.78
50	77.4 ± 1.02b	71.7 ± 1.26c	88.1 ± 1.31a	2.89
100	83.5 ± 2.32b	81.4 ± 1.63b	92.4 ± 1.16a	3.84
200	90.7 ± 1.97ab	88.8 ± 1.02b	95.8 ± 0.65a	4.65
IC_50_ (µg/g)	5.29 ± 0.32b	9.63 ± 1.04a	2.55 ± 0.84c	1.98

Data represent means ± standard error (*n* = 3). Significant differences (*P* ≤ 0.05) are indicated by different lowercase letters in the same column.

## Conclusion

This study demonstrates that supercritical CO₂ extraction pressure plays a decisive role in shaping the phytochemical composition and biological activities of *Heliotropium arbainense*. Extraction at higher pressure resulted in increased yield and enrichment of phenolic and flavonoid compounds, which was reflected in enhanced antimicrobial and anticancer activities, particularly against *Bacillus subtilis*,* Salmonella typhi*,* Candida albicans*, and SKOV3 ovarian cancer cells. In contrast, extracts obtained at lower pressure exhibited comparatively stronger anti-inflammatory activity, indicating that different pressure conditions may selectively favor specific bioactivities. These findings highlight the importance of pressure-dependent supercritical fluid extraction parameters to maximize the functional potential of medicinal plants.

While viable cells under SFE2 treatment (A3: 23.07%) were more abundant than under SFE1 (A3: 14.31%), SFE2 (low pressure) produced a slightly higher percentage of early apoptotic cells (A4: 43.79% vs. 43.39%) and a significantly larger necrotic population (A1: 21.83% vs. 13.29%).

From a biological perspective, the results confirm that *H. arbainense* is a promising source of bioactive compounds with multitarget in vitro activity, including antimicrobial, anti-inflammatory, and pro-apoptotic effects. The observed bioactivities are consistent with the phytochemical profile of the extracts, which is dominated by phenolic acids and flavonoids known for their antimicrobial and cytotoxic properties. Importantly, the study underscores that extraction strategy (not only plant species) critically determines biological performance. Despite these strengths, several limitations should be acknowledged. The biological evaluations were restricted to in vitro assays, and no in vivo validation or pharmacokinetic assessment was performed. In addition, while correlations between phytochemical composition and bioactivity were observed, direct molecular targets and mechanisms of action were not investigated. The antimicrobial analysis also revealed pathogen-specific variability, including the absence of activity against *Fusarium oxysporum*, which may reflect differences in compound solubility, extract composition, or strain sensitivity. Future studies should focus on extending these findings through in vivo models to confirm efficacy and safety, as well as mechanistic investigations to elucidate the molecular pathways underlying the observed antimicrobial and anticancer effects. Subsequent investigations should explore the refinement of supercritical extraction, particularly through the application of co-solvents, in order to improve the recovery of a wider range of bioactive compounds. Isolation and characterization of individual active compounds, followed by synergy and formulation studies, are also recommended to advance the pharmaceutical and biomedical potential of *H. arbainense*.

## Data Availability

All data generated or analyzed during this study are included in this published article.
